# Lymphatic Filariasis in Nigeria; Micro-stratification Overlap Mapping (MOM) as a Prerequisite for Cost-Effective Resource Utilization in Control and Surveillance

**DOI:** 10.1371/journal.pntd.0002416

**Published:** 2013-09-05

**Authors:** Patricia N. Okorie, George O. Ademowo, Yisa Saka, Emmanuel Davies, Chukwu Okoronkwo, Moses J. Bockarie, David H. Molyneux, Louise A. Kelly-Hope

**Affiliations:** 1 Institute for Advanced Medical Research and Training, College of Medicine, University of Ibadan, Ibadan, Nigeria; 2 Ministry of Health, Abuja, Nigeria; 3 Centre for Neglected Tropical Diseases – Liverpool School of Tropical Medicine, Liverpool, United Kingdom; Centers for Disease Control and Prevention, United States of America

## Abstract

**Background:**

Nigeria has a significant burden of lymphatic filariasis (LF) caused by the parasite *Wuchereria bancrofti*. A major concern to the expansion of the LF elimination programme is the risk of serious adverse events (SAEs) associated with the use of ivermectin in areas co-endemic with *Loa* filariasis. To better understand this, as well as other factors that may impact on LF elimination, we used Micro-stratification Overlap Mapping (MOM) to highlight the distribution and potential impact of multiple disease interventions that geographically coincide in LF endemic areas and which will impact on LF and vice versa.

**Methodology/Principal findings:**

LF data from the literature and Federal Ministry of Health (FMoH) were collated into a database. LF prevalence distributions; predicted prevalence of loiasis; ongoing onchocerciasis community-directed treatment with ivermectin (CDTi); and long-lasting insecticidal mosquito net (LLIN) distributions for malaria were incorporated into overlay maps using geographical information system (GIS) software. LF was prevalent across most regions of the country. The mean prevalence determined by circulating filarial antigen (CFA) was 14.0% (n = 134 locations), and by microfilaria (Mf) was 8.2% (n = 162 locations). Overall, LF endemic areas geographically coincided with CDTi priority areas, however, LLIN coverage was generally low (<50%) in areas where LF prevalence was high or co-endemic with *L. loa*.

**Conclusions/Significance:**

The extensive database and series of maps produced in this study provide an important overview for the LF Programme and will assist to maximize existing interventions, ensuring cost effective use of resources as the programme scales up. Such information is a prerequisite for the LF programme, and will allow for other factors to be included into planning, as well as monitoring and evaluation activities given the broad spectrum impact of the drugs used.

## Author Summary

Nigeria is estimated to have the highest burden of lymphatic filariasis (LF), a disease also known as elephantiasis, which is transmitted by mosquitoes and caused by the parasite *Wuchereria bancrofti*. The National LF Elimination Programme is planning to scale up the elimination programme through mass drug administration of ivermectin and albendazole. However, a major constraint to this expansion is the risk of serious adverse events (SAEs) associated with the use of ivermectin in areas co-endemic with *Loa*, the causative agent of tropical eye worm (loiasis). To better understand this and other factors that may impact on LF elimination, we collated and mapped all available LF data, and highlighted the overlaps with predicted loiasis prevalence distributions, onchocerciasis ivermectin treatment areas, and bed net distributions for malaria. This study provides a baseline overview for the LF Programme and will help to maximize existing disease interventions, ensuring cost effective use of resources as the programme scales up.

## Introduction

Lymphatic filariasis (LF) is one of the most debilitating neglected tropical diseases (NTD) in the world [1]. It is caused by the parasitic worms *Wuchereria bancrofti*, *Brugia malayi and B. timori* and is transmitted by *Anopheles, Culex, Aedes, Ochlerotatus* and *Mansoni* mosquitoes [1]. *Wuchereria bancrofti* is transmitted throughout the tropics in Africa, Asia, the Pacific and the Americas while *B. malayi* and *B. timori* are found in east and south Asia. The disease is endemic in 73 countries with an estimated 120 million people infected and 40 million people with clinical manifestations including lymphoedema (elephantiasis) of the limbs and urogenital disorders, especially hydrocele in men [2] [3]. In Africa, 34 countries are endemic, and Nigeria is believed to bear the highest burden of LF, with an estimated 80 to 120 million people at risk [3]–[5].

The Global Programme to Eliminate LF (GPELF) was launched in 2000 with the goal of eliminating LF as a public health problem by 2020 [1]. The principal elimination strategy is to interrupt transmission using Mass Drug Administration (MDA) with the combinations of albendazole plus ivermectin or albendazole plus diethylcarbamazine (DEC) administered once a year for at least five consecutive years. [1]–[3]. Overall, significant progress has been made, however, the scale up of programmatic activities has been slow in Africa, especially in countries with logistical challenges, conflict, instability and fragile infrastructures [6]. The wide and overlapping distribution of the filarial parasite *Loa* in Africa [7] is also a major impediment due to the risk of severe adverse events (SAEs) in co-infected individuals when treated with ivermectin [8] [9].

These constraints pose significant problems for the national LF programmes and GPELF with the potential to severely hinder the 2020 goal of LF elimination globally. To begin to address these complexities, a number of specific objectives and strategies have been developed. First, the GPELF strategic plan aims to achieve full geographical coverage with MDA by 2016, targeting the countries with the highest burden, including Nigeria [1]. Second, the use of integrated vector management (IVM) [10] is advocated in malaria co-endemic areas where both diseases are transmitted by *Anopheles* mosquitoes [11]. Finally, a provisional strategy for interrupting LF transmission in loiasis endemic countries recently developed recommends albendazole (400 mg) twice yearly in combination with vector control in all co-endemic areas [12]. Finally, mapping LF and *L. loa* at the lowest possible administrative unit is also considered important to identify small areas that can be treated for LF using the most appropriate regimes to reduce the risk of SAEs, which is considered to be highest when *L. loa* microfilaremia (mf) prevalence is ≥20%.

The coordinated effort of global disease control programmes is becoming increasingly important as many operate in the same countries and distribute interventions that have multiple benefits [13]–[16]. GPELF is likely to benefit from the activities of the Global Malaria Programme, including the recent scale up of insecticide treated/long-lasting insecticidal mosquito nets (ITNs/LLINs) and indoor residual spraying (IRS) [11]. These interventions have also been shown to impact LF transmission in a range of ecological settings [17], thus more synergy between the programmes in Africa could optimize resources and increase the impact on both diseases [15]–[18]. In countries such as Nigeria where malaria and LF are co-endemic and both transmitted by *Anopheles* mosquitoes [19] [20], the use of ITNs has shown to be effective at reducing LF transmission in *L. loa* co-endemic areas [21]. ITNs have also been successfully integrated with MDA activities in Central Nigeria with report of an increase in ITN ownership and retention [22] [23]. However, to take advantage of these programmatic links, more data on LF vectors is critical as there are many gaps in our knowledge as highlighted in the *Anopheles* database recently compiled for Nigeria [19].

Integrating activities and combining resources across the various NTD programmes will also have many advantages [13] [24]. For example, the African Programme for Onchocerciasis Control (APOC) has developed a sustainable community-directed treatment with ivermectin (CDTi) for the parasitic disease caused by the filarial worm *Onchocerca volvulus*, [25]–[27]. The CDTi approach has been successful in reaching millions of people across high transmission areas of onchocerciasis in Africa, and has also been used to distribute other health interventions including LF treatment and bed nets for malaria control [26]. Moreover, the maps of CDTi priority areas highlight the potential geographical overlap of onchocerciasis with LF, and it is likely that the wide and frequent use of ivermectin has reduced transmission in co-endemic areas [28]–[31]. However, the extent of this impact is yet to be determined at a large scale and needs to be quantified so that benefits from this and future NTD control programmes can be better understood and fully exploited [5] [32]–[34].

These issues are particularly relevant for Nigeria, given the large population at risk of *W. bancrofti* infection [3]–[5]. The National Lymphatic Filariasis Elimination Programme (NLFEP) is yet to complete LF mapping [3] [35] and will need significant financial and technical support to scale up MDA activities across this large, populous country. The aim of this paper, therefore, is to use the Micro-stratification Overlap Mapping (MOM) approach [24] to review and synthesize the current knowledge of the distribution of *W. bancrofti* in Nigeria, and factors that will impact on the control and elimination of LF such as loiasis co-endemicity, onchocerciasis control programmes, and malaria bed net distributions. This information is a prerequisite for effective planning and will help to optimize the future LF MDA implementation strategy to ensure safety, maximum cost effectiveness as well as impact.

## Methods

### Study location

**Figure 1:**
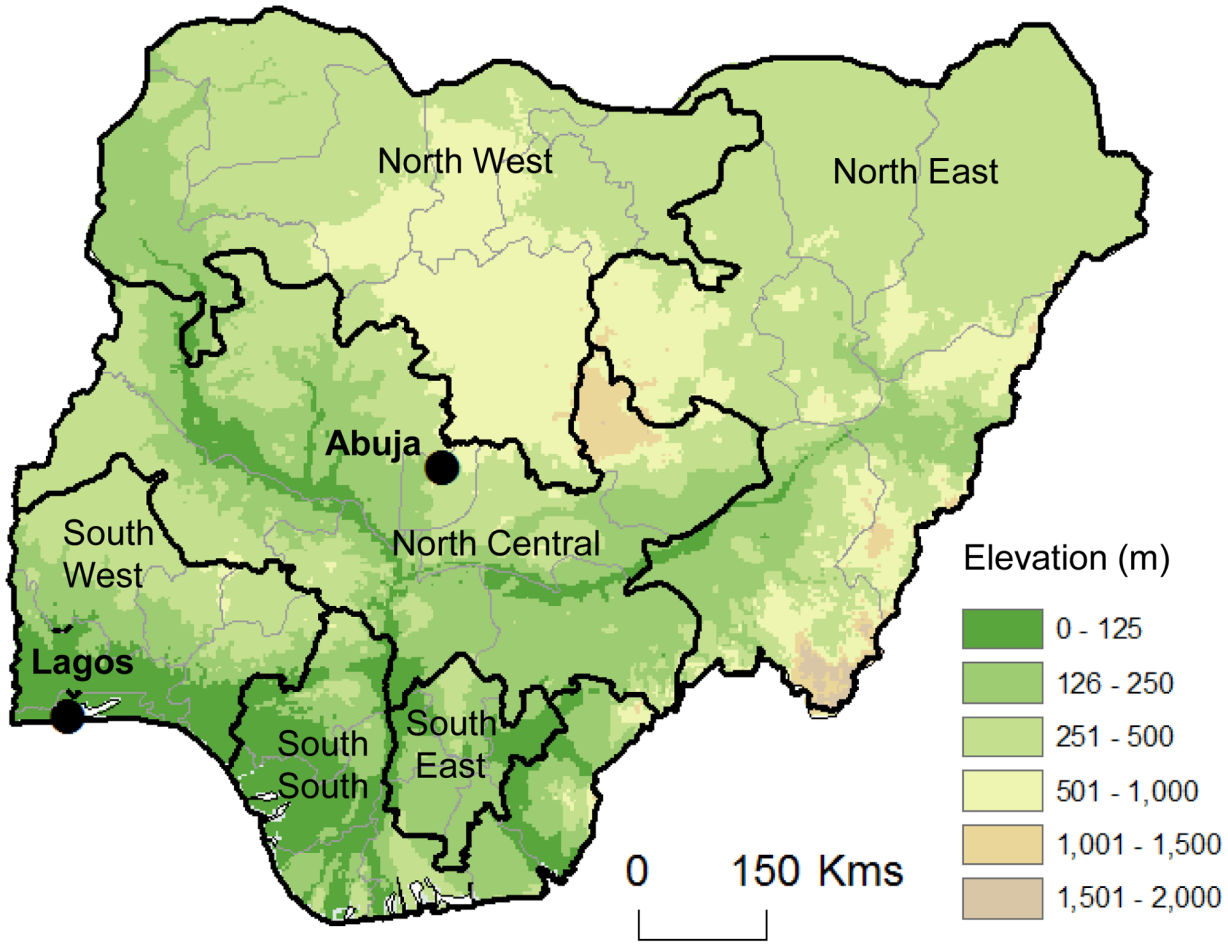
Map of Nigeria and its geopolitical zones. North Central - Benue, FCT, Kogi, Kwara, Nasarawa, Niger, Plateau. North East - Adamawa, Bauchi, Borno, Gombe, Taraba, Yobe. North West - Kaduna, Katsina, Kano, Kebbi, Sokoto, Jigawa,, Zamfara. South East - Abia, Anambra, Ebonyi, Enugu, Imo. South - Akwa-Ibom, Bayelsa, Cross-River, Delta, Edo, Rivers. South West - Ekiti, Lagos, Osun, Ondo, Ogun, Oyo. Note: Elevation data based on ETOPO2 global 2-minute gridded resolution from National Oceanic and Atmospheric Administration (NOAA) available from ESRI Redland, CA.

Nigeria is a Federal Republic comprising 36 States and its Federal Capital Territory, Abuja [35] [36]. The states are grouped into six geopolitical zones, the North Central (NC), North East (NE), North West (NW), South West (SW), South East (SE) and South (SS). Nigeria covers an area of approximately 923,768 sq. km, and has a large low plateau intersected by two major rivers, the Niger and Benue, in the central region of the country (Figure 1). It shares borders with Benin in the west, Chad and Cameroon in the east, and Niger in the north. Its coast in the south lies on the Gulf of Guinea on the Atlantic Ocean and Lagos, the former capital, is an important port city. Nigeria is Africa's most populous country with the total population estimated to be 160 million in 2012, with approximately 50% living in urban areas.

### LF prevalence data

To review and synthesize the current knowledge of the human distribution of LF in Nigeria, a systematic search for data in peer-reviewed published literature and national reports was carried out. The search was conducted using PubMed, JSTOR, Google, SCOPUS and other online scientific and historical databases. References were also obtained from the references listed within articles, and then from the references within those articles.

Studies and reports with data on the prevalence of i) LF infection as circulating filarial antigen (CFA) from using immunochromatographic tests (ICTs), antibodies by ELISAs, and microfilaria (Mf) from blood slides, and ii) disease cases (hydrocele, lymphodema) [2] [37] were identified and collated into a database. Information on the location/collection site (village, local government area (LGA) and State), and time period (month, year), was also collected for mapping and descriptive analyses. Specific information on whether MDA for LF had been administered prior to the LF prevalence measure was recorded and considered in the analysis. The range of methods used to detect LF in the different studies was recorded, as well as information on the mosquito species, which was cross-checked with the Nigerian *Anopheles* database [19].

The locations of the community or collection site were geo-referenced using the latitude and longitude coordinates obtained from references directly or by cross-checking the names with data from the GEOnet Names Server, Directory of Cities and Towns in the World databases [38] [39]. The coordinates of the midpoint of the LGA was used as a proxy for the locations that could not be allocated exact latitude and longitude coordinates. This is considered to be a limitation of the review and restricts any accurate detailed mapping. It is also acknowledged that LF prevalence distribution has a degree of bias as the data are based on the locations selected by the investigators in the original study, and does not take sampling methodologies between studies into account, which may affect the outcome.

In addition, selected data from the Federal Ministry of Health (FMoH) collected during LF mapping activities were collated and included in the database. The LF data available for this study were based on Mf prevalence rates collected in selected LGA sentinel sites during baseline surveys in 31 LGAs across 18 States of Nigeria. The WHO standard protocol was used to collect blood samples at night and examined for the presence of Mf. The coordinates of the midpoint of each LGA was used to map the LF prevalence. A national LF endemicity map by LGA was also available from the FMoH, which provided an overall CFA prevalence based on ICT survey in each State carried out between 2000 and 2010. Specific LGA data is not publicly available and not included in this database, however, the State-level information on the number of LGAs surveyed, prevalence range and year of survey is available in the recently published Master Plan for NTDs [35].

All the relevant information was entered into an Excel worksheet and data analysis was performed using Stata software (version 12, StataCorp, Texas, USA). All data were mapped using the geographical information systems (GIS) software ArcGIS 10.0 (ESRI, Redlands, CA) to produce maps of LF prevalence distributions, and to examine the geographical overlaps with loiasis-endemic areas, and the different intervention distributions.

### Loiasis co-endemicity

To examine the potential extent of LF and *L. loa* co-endemicity, the recent map of the predicted loiasis prevalence produced from a Rapid Assessment Procedure of Loiasis (RAPLOA) based on eye worm history carried out between 2004 and 2010 across Africa, including Nigeria [7], was imported into ArcGIS. Three levels of predicted loiasis prevalence were digitised (i.e. outlined, shaded) based on the defined distribution boundaries, which included low <20%, medium 20–40% and high >40% prevalence areas; the latter is equivalent to mf prevalence of >20%. The different levels of loiasis prevalence and the overlap with LF prevalence distributions were highlighted to help identify potential low risk (i.e. loiasis <20%) and medium to high risk (i.e loiasis >20%) SAE areas.

### Interventions overlap maps

#### Onchocerciasis control programmes

To examine the distribution of ivermectin and its association with LF and loiasis endemicity, the CDTi map produced from REMO surveys carried out in 2004 and 2005 [31] was imported into ArcGIS. The CDTi priority (i.e. ivermectin treatment) areas were digitized, and the overlap with LF and loiasis distributions highlighted. It is acknowledged that the CDTI priority areas do not identify the specific location, frequency and duration of ivermectin distribution, but provide a broader overview of potential treatment areas. Detailed data on treatment areas are not publicly available.

#### Malaria bed net distributions

To examine the potential impact of vector control in co-endemic areas, data on LLIN coverage based on the percentage of households with at least one LLIN for each geopolitical zone was obtained from the Malaria Indicator Survey (MIS) carried out in 2010. The LLIN coverage rates were based on data collected from 7200 households in 12 states across all geopolitical zones [40]. Coverage rates by each zone were mapped based on three levels, which included <25%, 25–50% >50%. The different levels of LLIN coverage and overlap with LF and *L. loa* co-endemic areas were highlighted to help identify areas that could potentially benefit from this intervention.

## Results

### LF data summary

**Figure 2:**
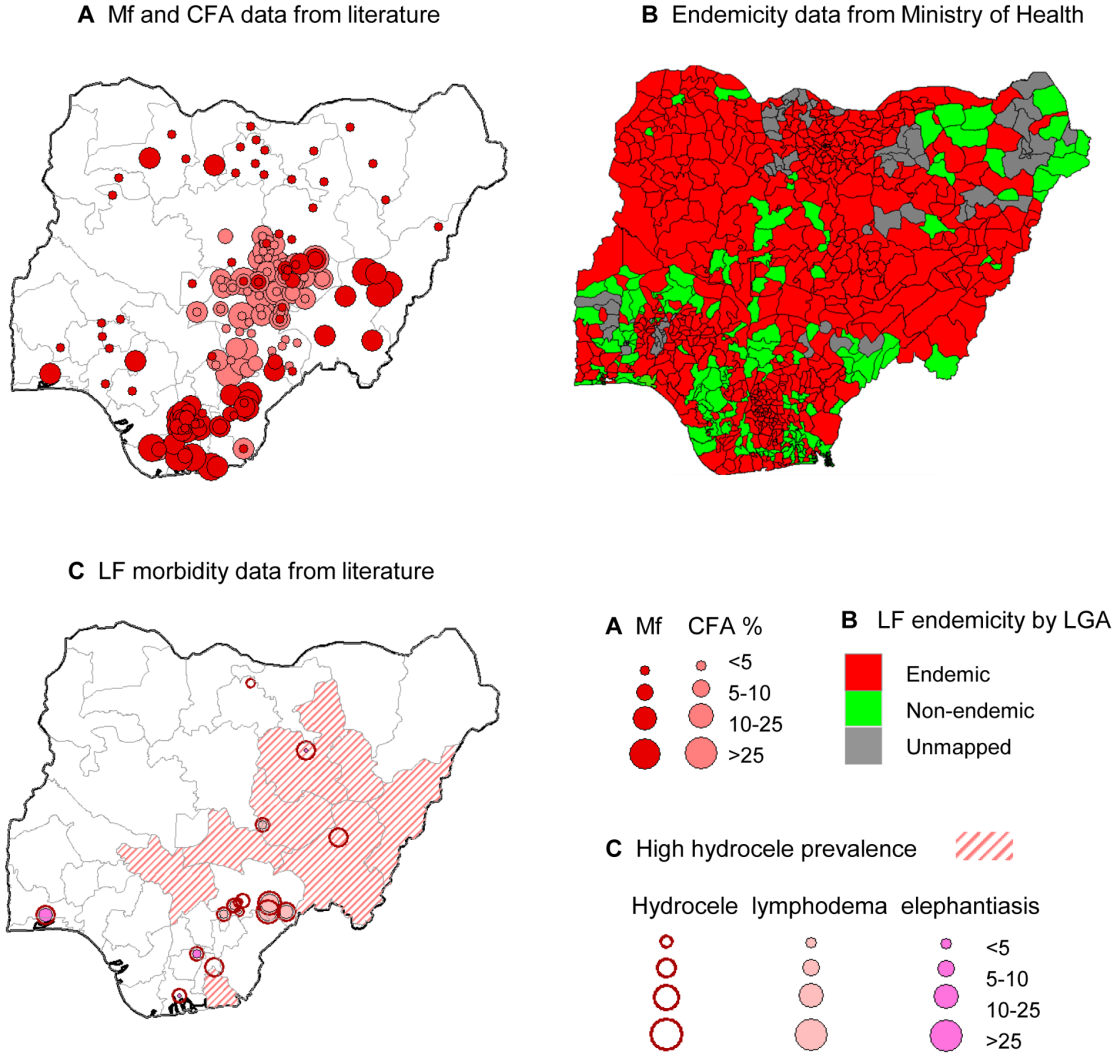
LF prevalence data, endemicity status and disease data. **a**. CFA and Mf data. **b**. LF endemicity. **c**. Disease data. Note: Data source for CFA, MF prevalence (2a) and disease (2c) data available in Table S1. LF endemicity map (2c) developed by FMoH.

In total, 41 studies [41]–[81] from 68 published and unpublished filariasis studies identified in the literature were found to have examined the prevalence, clinical manifestations and entomological aspects of LF in Nigeria (Table S1). The studies excluded from the review had reported data on *L. loa* and/onchocerciasis only. The majority of LF studies (n = 30) were conducted post 2000 [41]–[69], nine studies were conducted between 1980 and 2000 [70]–[78], and three studies were conducted pre 1980 [79]–[81]. The studies indicate that LF is present in 19 States across all six geopolitical zones of the country (Figure 2a). The majority of studies were from the NC geopolitical zone, with the most comprehensive studies carried out in Plateau and Nassarawa States [49] [53] [54] [63] [66].

The FMoH sentinel site Mf prevalence data were carried out more widely in 31 LGAs across 18 States. All information was added to the database (Table S1) and mapped with the other specific Mf data described above (Figure 2a). The FMoH national endemicity map indicated that out of the 774 LGAs in Nigeria, 541 were classified as endemic, 164 were classified as non-endemic and 69 remained to be mapped (Figure 2b). The related state-level data are found in Nigeria Master Plan for NTDs [35].

The range of methods used to detect the presence of LF in Nigeria included serological methods (using ICTs or ELISA), parasitological methods (blood films for Mf) and physical examination for clinical manifestations (lymphodema, hydrocele), and were used either alone or in combination. Studies carried out before the 1980s only used parasitological examination of blood films, whereas post 2000, a combination of serology and parasitological methods were most widely used (Table S1).

### LF prevalence distribution

In total there were 258 individual data points from 152 unique locations where the prevalence of *W. bancrofti* was measured by CFA or Mf. The average CFA and Mf rates by State are summarized in Table 1 and 2. The overall mean CFA prevalence rate across the country was 14.0% (n = 134; range 0% to 66.0%), and the overall mean Mf prevalence rate was 8.2% (n = 162; 0 to 47.4%). The CFA and Mf prevalence rates by geopolitical zones indicate that the highest rates occur in the NC, NE, NW and SS zones. The highest CFA prevalence rate recorded was 66% recorded at Ogi-Utonkon, Ado LGA, Benue State, NC [64], while the highest Mf rate was 47.4% recorded at Zing LGA, Taraba State, NE [55] (Table S1).

**Table 1:** Summary of CFA prevalence by state.

Zone and State	No. of sites	No. of persons tested	Mean (%)	95% CI (lower)	95% CI (Upper)
**North Central**					
Benue	23	2189	12.9	11.5	14.4
Nassarawa	46	19026	11.4	11.0	11.9
Plateau	61	27325	16.5	16.1	16.9
Plateau, Nassarawa	1	4120	22.5	21.2	23.8
**North West**					
Kaduna	1	341	10.0	7.0	13.7
**South**					
Bayelsa	1	1803	11.3	9.9	12.9
Cross River	1	222	17.0	12.4	2.7
**Total**	**134**	**55026**	**14.0**	**13.7**	**14.3**

Note: All data included i.e. number of MDA rounds not taken into account.

**Table 2:** Summary of Mf prevalence by state.

Zone and State	No. of sites	No. of persons tested	Mean (%)	95% CI (lower)	95% CI (Upper)
**North Central**					
Benue	3	1903	7.0	5.9	8.2
FCT*	1	-	0.0	-	-
Kogi*	1	-	0.0	-	-
Kwara*	1	-	0.0	-	-
Kwara, Kogi, Plateau	1	172	13.9	9.1	20.0
Nassarawa	21	4431	1.1	0.8	1.5
Niger*	1	-	3.8	-	-
Plateau	30	6322	3.4	3.0	3.9
**North East**					
Adamawa*	1	-	1.2	-	-
Bauchi	4	4114	1.2	0.8	1.5
Taraba	7	3966	23.5	22.2	24.9
Yobe*	3	-	0.0	-	-
**North West**					
Jigawa*	3	-	0.3	-	-
Kaduna*	1	-	0.3	-	-
Kano	6	180	1.0	0.1	4.0
Katsina	1	257	22.6	17.6	28.2
Kebbi*	1	-	0.0	-	-
Zamfara*	3	-	4.6	-	-
**South East**					
Anambra*	1	-	18.8	-	-
Ebonyi	1	1243	16.9	14.9	19.1
Imo	39	9131	12.8	12.1	13.5
Imo/Anambra	5	500	16.0	12.9	19.5
**South South**					
Akwa Ibom*	1	-	17.6	-	-
Bayelsa	2	2583	18.0	16.5	19.5
Cross River	11	1903	10.2	8.9	11.6
Edo*	1	-	2.2	-	-
Rivers	5	2837	24.2	22.6	25.8
**South West**					
Ekiti*	1	-	1.2	-	-
Ogun	1	317	17.0	13.1	21.6
Ondo*	3	-	5.6	-	-
Osun*	1	-	1.8	-	-
Oyo	1	915	3.4	2.3	4.8
**Total**	**162**	**40775**	**8.2**	**7.9**	**8.5**

Note: All data included i.e. number of MDA rounds not taken into account.

* Value for number of persons tested not available.

Confidence intervals calculated in Stata software (version 12, StataCorp, Texas, USA).

**Figure 3:**
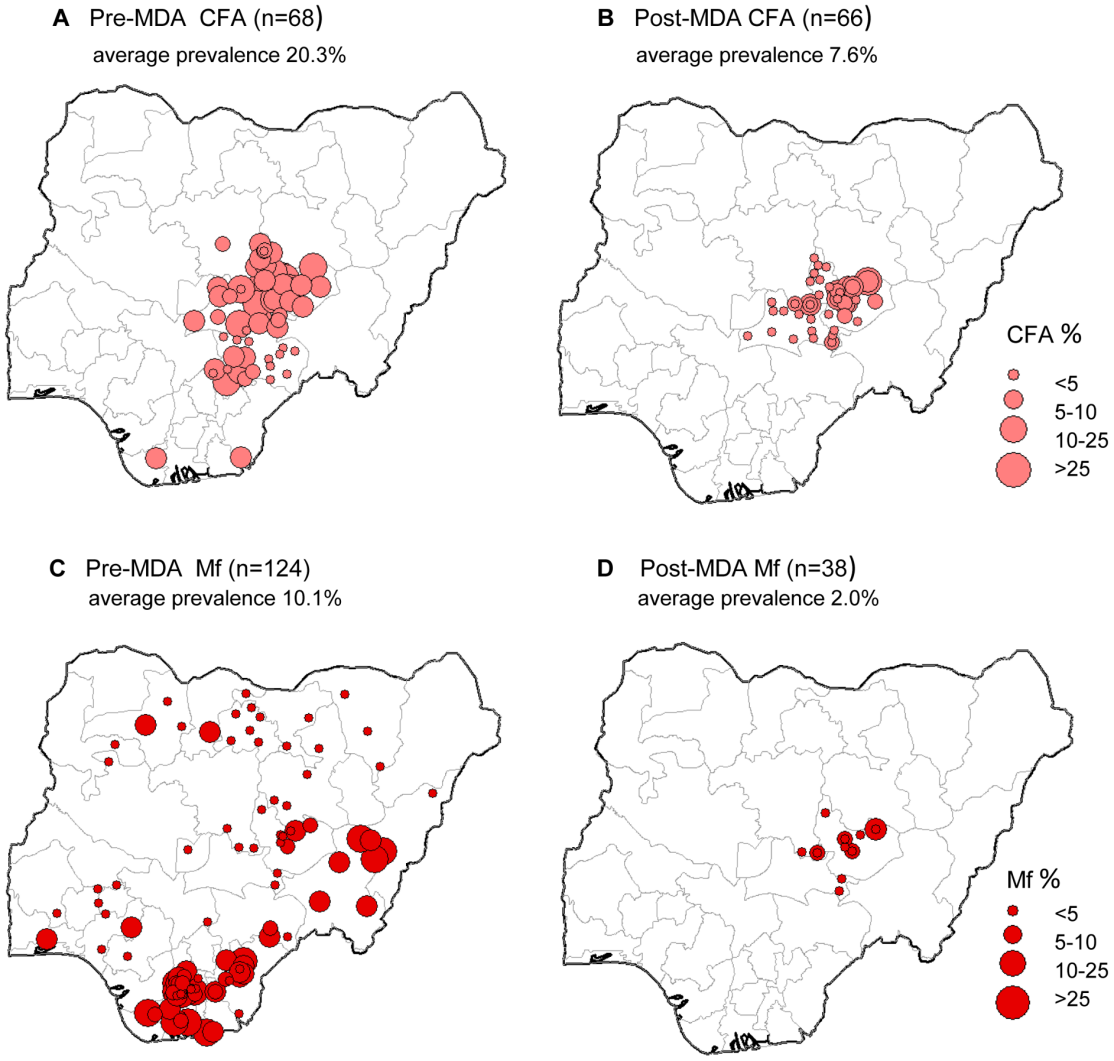
LF prevalence data pre-MDA and post-MDA. **a**. Pre-MDA CFA (n = 68). **b**. Post-MDA CFA (n = 66). **c**. Pre-MDA Mf (n = 124). **d**. Post-MDA Mf (n = 38). Note: Data source for CFA, MF prevalence available in Table S1.

Overall, there were marked differences in *W. bancrofti* prevalence at sites that had not received MDA i.e. pre-MDA, compared to those sites that had received MDA i.e. post-MDA. The average CFA prevalence in pre-MDA sites, was 20.3% (n = 68; range 0% to 66.0%), which was approximately 3 times higher than the average CFA prevalence in post-MDA sites, 7.6% (n = 66, range 0.2% to 31.5%) (Figure 3a,b). The average Mf prevalence in pre-MDA sites was 10.1% (n = 124; range 0% to 47.4%), which was approximately 5 times higher than the average Mf prevalence in post-MDA sites, 2.0% (n = 66, range 0% to 12.1%) (Figure 3c,d). The distribution of post-MDA sites occur in the two States of Plateau and Nassarawa, and are the result of an extensive MDA programme delivering the combination of ivermectin and albendazole as detailed in the study publications [49] [53] [54] [63]. Baseline LF mapping was conducted in 1999 and 2000 in 30 LGAs of the two States, and MDA launched in 2000, and monitored from 2000 to 2009. Details are contained in the specific reference [63] (Table S1).

### Clinical manifestations

The most extensive study on clinical manifestations was conducted by Nwoke *et al.* [82], who used hydrocele as a clinical marker to estimate LF prevalence. A rapid epidemiological mapping survey (REM-LF) was conducted across 25 States and 536 villages in Nigeria. Details of the specific study sites are not available, however, the survey found that hydrocele was absent in 339 (63.3%) villages, and present in 197 (36.8%) villages, which were found to have different levels of hydrocele severity. Hydrocele was absent in Jigawa and Kano (NW), and Ogun (SW) States. Very few hydrocele cases (1–3%) were found in northern Borno (NE), Kaduna and Zamfara (NW), Edo (SS), Imo (SE),and in Ekiti, Ondo, Osun, Oyo (SW) States. The highest hydrocele rates were found in the NE States of Adamawa, Bauchi, Gombe, Taraba and southern Borno, in the NC states of Kogi, Plateau, Nassarawa, and in the northern part of Akwa Ibom State in the SS (Figure 2c) [82].

The clinical signs that were reported included limb lymphodema, hydrocele. chyluria and elephantiasis, and were from a few specific areas of the country [45]–[49] [59]–[62] [65] [68]–[71] [74]–[75] [77]. For hydrocele, there were 22 sites with prevalence data ranging from 0.1% to 50%, while for lymphodema, 12 sites recorded prevalence rates ranging from 1% to 49%. The prevalence of limb elephantiasis was also recorded in 5 sites, which ranged from 1.7% to 11.8%. The distribution of these study sites is shown in Figure 2c, together with the high hydrocele prevalence states described by Nwoke et al. [82], and highlight the geographical concordance with CFA and Mf distributions which occur in the central south and eastern regions of the country (Figure 2a).

### LF and loiasis co-endemicity

**Figure 4:**
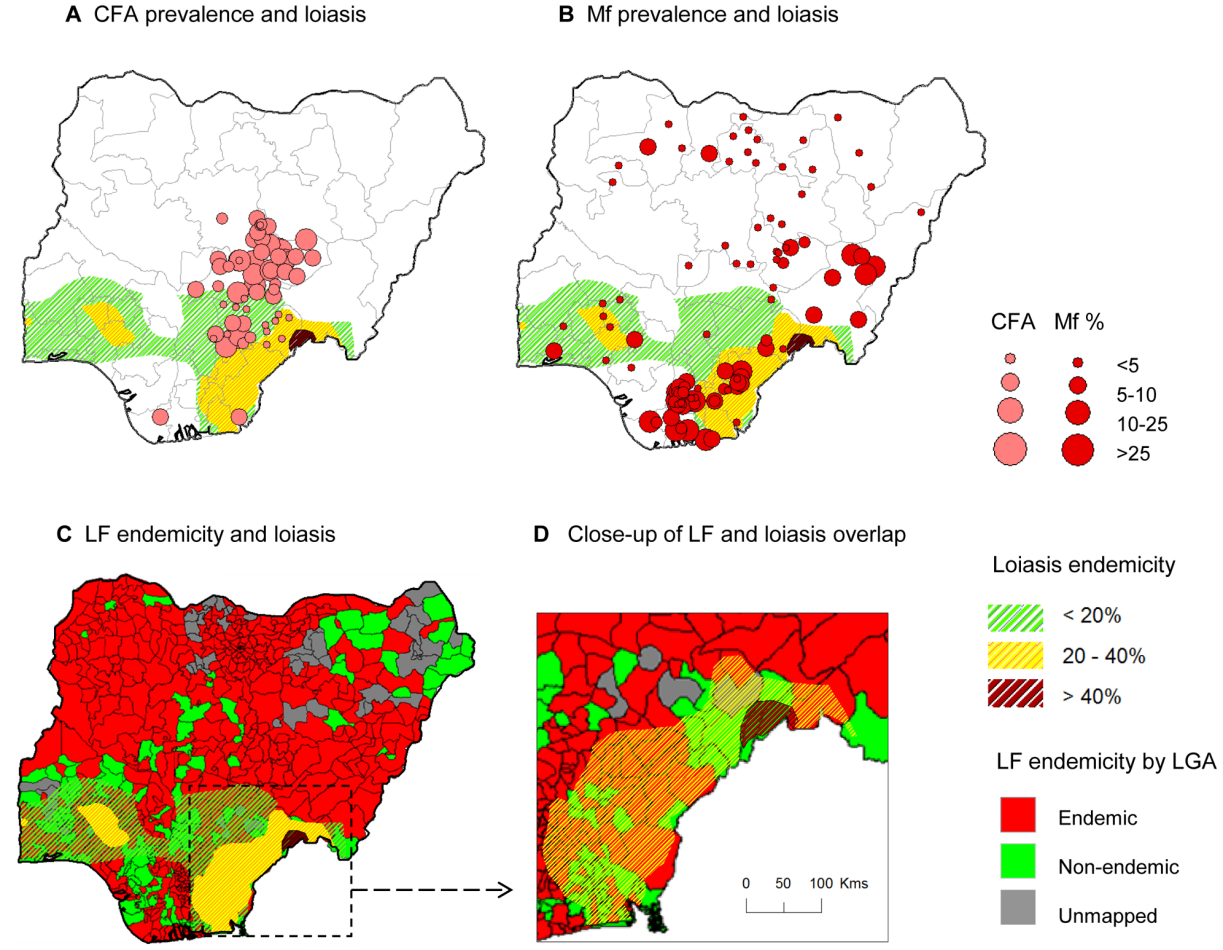
LF prevalence overlapping loiasis areas. **a**. CFA prevalence and loiasis. **b**. Mf prevalence and loiasis. **c**. LF endemicity and loiasis. **d**. Close up of LF and loiasis overlap. Note: Loiaisis endemicity based on eye worm history map determined from RAPLOA surveys published by Zouré et al. 2011 [7]. Three levels of loiasis shaded green <20%, yellow 20–40% and dark brown >40% highlight the extent of geographical overlap between CFA (4a) and MF (4b) prevalence data points available in Table S1. Figure 4c shows loiasis overlap with LF endemicity map developed by the FMoH, and the medium to high risk loiasis areas (yellow and dark brown shading) of the south eastern region is shown close-up in 4d. The small localized high risk loiasis area (dark brown) geographically coincides with areas classified as LF non-endemic (solid green).

LF prevalence was examined in relation to the *L. loa* distribution in Nigeria defined by the RAPLOA surveys reporting eye worm history, which were carried out in 381 villages between 2002 and 2010 [7]. Loiasis was found predominately in the southern region of the country, with the highest risk in east along the border with Cameroon, which had a localized area >40% in the States of Taraba and Benue (Figure 4). Overall, there was minimal geographical overlap with the number of LF prevalence sites determined by CFA. The majority of sites with medium to high LF prevalence rates >25%, were found in low loiasis prevalence areas (<20%) where the risk of SAEs are considered to be low (Figure 4a). Similarly, there was minimal geographical overlap with the number of LF prevalence sites determined by Mf, however, more sites with medium to high LF prevalence rates >25% were found in medium loiasis prevalence areas (20–40%) where the risk of SAEs is potentially high (Figure 4b). The overlap with the LF endemicity map available from the FMoH shows a combination of endemic and non-endemic LGAs in the low (<20%) to medium (20–40%) loiasis prevalence areas (Figure 4c). Only LF non-endemic LGAs were found in the high risk loiasis area (>40%), which is highlighted in the close up of the map in Figure 4d.

### Intervention overlap maps

**Figure 5:**
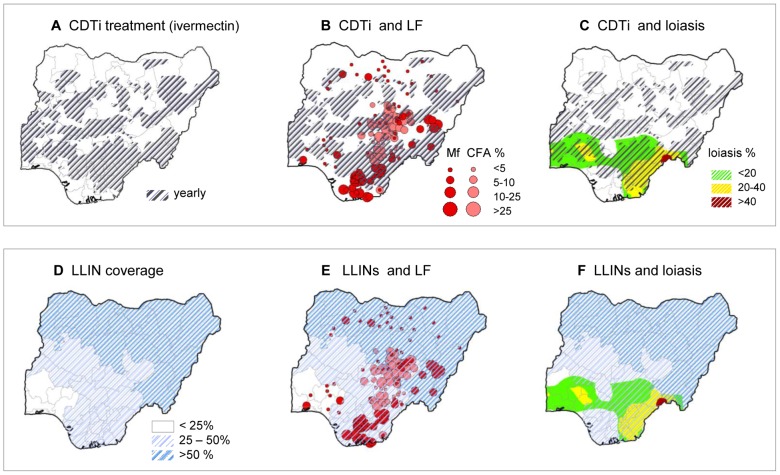
LF prevalence and intervention distribution overlap. **a**. CDTi treatment (ivermectin). **b**. CDTi and LF. **c**. CDTI and loaisis. **d**. LLIN coverage. **e**. LLINs and LF. **f**. LLINs and loiasis. Note: Data source for CDTi (5a) based on WHO-APOC Country profile – Nigeria [31] (shaded grey) and for LLIN coverage (5d) based on Malaria Indicator Survey [40] (shaded blue) to highlight the geographical overlap with CFA and Mf data prevalence points from Table S1(5b and 5e) and loiasis map by Zouré et al. 2011 [7] (5c and 5f) respectively.

The onchocerciasis CDTi priority areas [31] are shown in Figure 5a, and illustrate that large areas across the central region of the country are being targeted with ivermectin treatment. Since 1992, ivermectin has been distributed annually to 80% of the total population at risk, estimated at ∼38,331,140 people in 430 endemic LGAs [35]. The LF endemic areas to potentially benefit from CDTi priority areas are extensive and include large areas of NC, NW and NE zones of the country. The LF programme could readily add albendazole to the ivermectin being distributed in these areas (Figure 5b). The potential risks associated with ivermectin treatment for *O. volvulus* are related to potential SAEs in areas where *W. bancrofti* and *L. loa* are co-endemic in the southern region of the county, especially in the States of Benue, Cross River, Ebonyi, Enugu, Osun, Ekiti and regions of Edo, Ondo and Ogun States (Figure 5c).

The distribution of LLIN coverage across the six different geopolitical zones is shown in Figure 5d. The highest LLIN coverage occurred in the NE (61.8%) and NW (58.2%) zones, followed by the SS (43.5%), SE (32.1%), NC (32.1%) and SW (20.3%) zones respectively. This shows that the highest LLIN coverage occurred in the northern region of the country where LF does not appear to be highly endemic (Figure 5e). The lowest LLIN coverage occurred across the southern region of the country, which coincides with areas where both LF and loiasis are considered endemic and co-endemic (Figure 5f).

## Discussion

The database and series of maps produced in this study provide critical information to the Nigerian National NTD Programme. The LF Programme, specifically, will benefit as the implementation of MDA will be more effective in the coming years as it scales up to reach national coverage with support from the government and international stakeholders. This database represents the largest geo-referenced source of data on *W. bancrofti* publicly available for any country in Africa, and provides a basis on which to add future programmatic and research data collected from routine activities and systematic surveys in Nigeria over time. Importantly, it highlights the few selected studies and large data gaps on the burden of disease, which is an increasingly important area for GPELF and the international community given the significant social stigma and economic consequences of this disabling disease [2]. This database also complements the extensive *Anopheles* database recently developed by Okorie et al. [19], which comprises all available information on the main vectors of malaria and LF in Nigeria. The combination of the two databases will help to prioritise programmatic activities by appropriately targeting high LF prevalence areas, and by identifying areas with data gaps where more information is needed.

The LF prevalence data and maps clearly show the widespread endemicity across the country, and the need to implement MDA on a large scale with high levels of coverage. This will require significant support as currently only 18,591,932 (17.5%) of the targeted population are receiving MDA with ivermectin and albendazole, with varying levels of coverage [35]. The impact of MDA on LF transmission in most of the States and LGAs is yet to be determined as this intervention has only started to expand. However, detailed studies carried out in the States of Plateau and Nassarawa have highlighted the effectiveness of this intervention and the importance of documenting impact and progress through systematic monitoring and evaluation [53] [63]. These results suggest that Nigeria could significantly reduce *W. bancrofti* transmission in the next few years with a collective effort and commitment through increased human and financial resources from both national and external donors and stakeholders. For example, the concerted efforts geared towards the control of onchocerciasis by APOC have made a huge impact on the population by markedly reducing the prevalence and morbidity associated with the disease [83].

The LF programme also needs to take advantage of the interventions already being distributed by other programmes that could enhance its efforts towards elimination. This study highlights that large areas of Nigeria are CDTi priority areas and have received multiple rounds of ivermectin for the control of *O. volvulus* over the past decade [31] [34]. It is likely that ivermectin has already impacted on LF as shown elsewhere in West Africa [28] [29], and possibly interrupted transmission in low prevalence areas, especially those that have also received LLINs for malaria control. More detailed studies examining the impact of ivermectin on LF in Nigeria are important to fully appreciate its role in reducing prevalence levels. The CDTi priority areas also provide an opportunity for the LF programme to add albendazole to the current ivermectin regimes, which will save time and lead to considerable cost savings given the shared use of human resources and infrastructure. Further, the collation and examination of intervention data at State and LGA level using the MOM approach [24] will help produce finer scale maps informing the regional and local programmes of the risk and benefits, and actions that need to be taken. It will also help determine if other NTDs, such as the soil-transmitted helminths (STH), need to be considered.

To date only mebendazole has been used for STH in Nigeria, however, albendazole is being introduced in addition to medendazole in 2013 by the FMoH and is also distributed during child health weeks, thus providing some indirect treatment in LF endemic areas [35]. Integrating activities with the STH programme will be essential as the expansion of the LF Programme will also provide treatment for STH and support for the new STH programme. Importantly, if albendazole is introduced and scaled up for LF in the loiasis endemic regions the impact on both diseases may be significant. Taking these factors into account is important in a large, ecologically diverse and populous country like Nigeria, where *W. bancrofti* is co-endemic with other diseases and varies considerably in the different geopolitical zones and ecological settings [20] [32]–[35]. This variability requires that different intervention strategies should be developed and implemented consonant with the most up to date information and guided by WHO guidelines.

Nigeria, unlike several other *L. loa* endemic countries, has not reported any SAEs associated with ivermectin treatment for *O. volvulus*, which is shown to cover more than 90% of the *L. loa* area [35]. The reason for this is unclear but may be related to the different levels of endemicity and the strategies that have successfully been used to address the risk [31]. It may also be related to the extent of geographical overlap in high risk *L. loa* areas, which appears to be less than countries in Central Africa such as Cameroon and the Democratic Republic of Congo, where SAEs have had a major negative impact on NTD programmes [7]–[9]. In Nigeria, LF and *L. loa* appear to be co-endemic at low to medium prevalences (<40%) and only in the southern region of country. This information will help focus the distribution of the current WHO recommended alternative strategy of twice yearly treatment albendazole and distribution of ITN/LLINs [12]. Coordination with STH programmes will be essential, especially as new funding becomes available and programmes expand into co-endemic areas [31] [32]. In the future new alternative drugs and regimes such as the doxycycline an anti *Wolbachia* ' macrofilaricide or adult sterilising agent [84]–[86], may become available for co-endemic area and fine scale mapping will help to identify those populations most appropriate to target.

Strengthening linkages with the National Malaria Control Programme will be crucial, especially as there has been significant scale up of bed net distribution through a new campaign since previously reported ITN coverage in 2005 [87] and the LLIN coverage in 2010 reported here [88]. In mid-2012, nearly 50 million LLINs were reported to have been distributed across the country representing 73% of the total number expected for universal coverage distribution. [89], [90]. This dramatic increase in vector control has major implications for LF as bed nets have shown to impact on *W. bancrofti* transmission, especially in *Anopheles*-transmitted areas [17] [21] [91] [92]. Moreover, entomological data from a longitudinal study conducted in South East Nigeria has showed that full coverage with LLINs can interrupt LF transmission even in the absence of MDA [21]. More information on *Anopheles* vectors and their susceptibility to insecticides being used in vector control is urgently needed [19]. The role of xenomonitoring should also be considered as it may be more reliable and cost effective in the long term, especially in Nigeria where there is a wide range of existing entomological capacity and expertise that could be specifically developed and strengthened to help monitor the elimination of the disease.

### Conclusion

This MOM work builds on the recent study carried out in Democratic Republic of Congo [24], which first used the new overlap mapping approach to collate and map all available country data on *W. bancroft*i, examine the extent of *L. loa* co-endemicity and determine the risk and benefits of different intervention strategies. Collectively the two studies address two important countries in Africa with respect to the elimination of LF, as they have the highest burdens of disease, collectively accounting for more than 170 million people at risk. Furthermore, their LF Programmes are yet to scale up to reach full national MDA coverage taking into account the co-endemic areas of *L. loa*, which may require alternative treatment strategies in selected areas, and coordination with other NTD elimination and malaria control programmes. We advocate that the MOM approach should be used more widely over time and space, and at different geographical scales to better monitor and understand the impact of single and multiple interventions, and to assess progress towards elimination of LF and other diseases. Such an approach is also necessary for national planning purposes as well as increasing the cost effectiveness and coordination of programmes where different strategies are deployed, and where there have been previous interventions which will impact on the goals of the LF programme.

## Supporting Information

Checklist S1 PRISMA checklist.
(DOC)

Flowchart S1 PRISMA flowchart.
(DOCX)

Table S1 LF prevalence database.
(XLSX)
