# Supplementary material for: Lymphatic Filariasis in Nigeria; Micro-stratification Overlap Mapping (MOM) as a Prerequisite for Cost-Effective Resource Utilization in Control and Surveillance
Source: PLoS Negl Trop Dis. 2013 Sep 5;7(9):e2416. doi: 10.1371/journal.pntd.0002416 (PMC3764235; doi:10.1371/journal.pntd.0002416)
Supplement: Flowchart S1 — PRISMA flowchart. (DOCX) [file pntd.0002416.s002.docx]

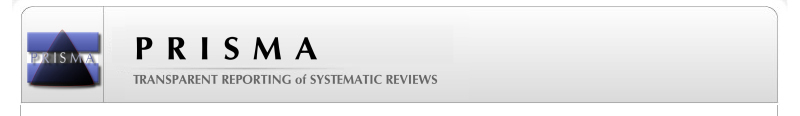
**PRISMA 2009 Flow Diagram**

Studies included in quantitative synthesis (meta-analysis)
(n = 41 )

Studies included in qualitative synthesis
(n =41 )

Full-text articles excluded, with reasons
(n = 27 )

Full-text articles assessed for eligibility
(n = 68 )

Records excluded
(n = 0 )

Records screened
(n = 68 )

Records after duplicates removed
(n = 68 )

Additional records identified through other sources
(n = 37 )

## Identification

## Eligibility

## Included

## Screening

Records identified through database searching
(n = 47 )
